# Shaofu Zhuyu decoction for primary dysmenorrhea: A protocol for systematic review and meta-analysis

**DOI:** 10.1097/MD.0000000000026226

**Published:** 2021-06-11

**Authors:** Lei Yuan, Dan Lin, XueLiang Kang, Yu Ye

**Affiliations:** College of Basic Medicine, Guizhou University of Traditional Chinese Medicine, Guiyang, Guizhou, China.

**Keywords:** meta-analysis, modified Shaofu Zhuyu decoction, primary dysmenorrhea, protocol, Shaofu Zhuyu decoction, systematic review

## Abstract

**Background:**

Primary dysmenorrhea (PD), one of the common gynecological diseases, is more common in adolescent women. According to epidemiological investigation, the incidence of PD accounts for about 60% of all patients with dysmenorrhea, and its symptoms seriously affect the normal working life of women, so it is important to find a more safe and effective treatment. In recent years, traditional Chinese medicine has made a lot of achievements in the treatment of female PD, among which a number of clinical studies have shown that Shaofu Zhuyu decoction (SFZY) can significantly improve the symptoms of dysmenorrhea and improve the therapeutic effect. Therefore, we intend to conduct a systematic review to further clarify the efficacy and safety of SFZY in the treatment of PD.

**Methods:**

We will search each database from the built-in until May 2021. The English literature mainly searches Cochrane Library, PubMed, excerpt medica database, and Web of Science, while the Chinese literature comes from China National Knowledge Infrastructure, Chinese biomedical literature database, VIP, and Wanfang database. Simultaneously we will retrieval clinical registration tests and grey literatures. This study only screen the clinical randomized controlled trials about XFZY for PD to assess its efficacy and safety. The 2 researchers worked independently on literature selection, data extraction, and quality assessment. The dichotomous data is represented by relative risk, and the continuous is expressed by mean difference (MD) or standard mean difference, eventually the data is synthesized using a fixed effect model or a random effect model depending on whether or not heterogeneity exists. The clinical efficacy and the score of dysmenorrhea symptoms were evaluated as the main outcomes. The artery pulsatility index (API), resistance index (RI), peak systolic/diastolic peak (S/D) were secondary outcomes. Finally, meta-analysis was conducted by RevMan software version 5.3.

**Results:**

This study will provide high-quality evidence for treatment of PD with SFZY in terms of effectiveness and safety.

**Conclusion:**

This systematic review aims to provide new options for SFZY treatment of PD in terms of its efficacy and safety.

**Ethics and dissemination:**

This study does not require ethical approval. We will disseminate our findings by publishing results in a peer-reviewed journal.

**OSF registration number:**

DOI: 10.17605/OSF.IO/DXZHR.

## 1 Introduction

Primary dysmenorrhea (PD), also known as functional dysmenorrhea due to organic lesions without reproductive organs, is characterized by periodic abdominal pain during or before and after menstruation, and may be accompanied by headache, fatigue, dizziness, nausea, vomiting, diarrhea, and other discomfort. Severe pain may even occur fainting.^[1]^ At present, the pathogenesis of PD is not clear. Modern medicine believes that its etiology is mainly related to neurological, endocrine, immune, genetic, and other factors. At present, it is mainly treated with painkillers, such as prostaglandin synthase inhibitors or oral contraceptive, but these drugs are only for the purpose of relieving pain, and it is not easy to adjust the imbalance of patients’ endocrine system as a whole, and there are many adverse reactions in patients. It is not easy to be accepted by the majority of patients.

Traditional Chinese medicine treatment of PD has obvious curative effect in relieving pain and improving concomitant symptoms, with little side effects and long-lasting effect, so it has become one of the dominant diseases in the treatment of traditional Chinese medicine. According to traditional Chinese medicine, the causes of dysmenorrhea are mainly divided into “normal deficiency of essence and blood, sharp changes in qi and blood during menstruation” and “pain if not proud” and “obstruction of evil qi and blood.”^[2]^ In the treatment, regulating blood circulation and relieving pain during menstruation as the main treatment principle, activating blood circulation and removing blood stasis during the intermenstrual period and regulating qi and blood circulation are the main principles. Shaofu Zhuyu decoction (SFZY) can be selected for clinical treatment. SFZY comes from Wang Qingren compound prescription of Yilin Correction in Qing Dynasty, which is composed of fennel, dried ginger, Rhizoma Corydalis, Ligusticum chuanxiong, Angelica sinensis, and so on.^[3]^

The compound of SFZY contains a variety of chemical components, including glycosides, coumarins, flavonoids, volatile oils, organic acids, and alkaloids. Its effect is promoting blood circulation and removing blood stasis, warming menstruation, reducing swelling, and relieving pain.^[4]^ Modern pharmacological studies have shown that SFZY can significantly reduce blood TXA2, and increase PGI2, thus increasing its effect of promoting blood circulation and removing blood stasis.^[5]^ Its total flavonoids and organic acids in Puhuang can inhibit platelet aggregation induced by ADP, arachidonic acid and collagen in vivo and in vitro, and its decoction can inhibit thrombosis and improve hemorheological parameters.^[6,7]^ Significantly inhibit platelet aggregation and antithrombotic effect induced by ADP and collagen.^[8]^ Some studies also have proved that the compound can significantly reduce the contraction and relaxation intensity of uterine spontaneous movement and has a good antispasmodic effect.^[9]^ In clinic, SFZY can regulate the secretion of prostaglandin, inhibit the spastic contraction of uterine smooth muscle, increase blood flow, improve local ischemia and hypoxia, thus exerting its short-term and long-term analgesic effect.^[10]^

To sum up, no matter from a large number of animal experiments or clinical experiments, it has been proved that SFZY has a very definite effect in the treatment of PD. However, there is no systematic study on the efficacy and safety of SFZY in the treatment of chronic pelvic inflammation. In this study, meta-analysis method was used to systematically evaluate the efficacy and safety of SFZY in the treatment of PD, and to provide strong evidence-based medicine support for its clinical application.

## 2 Methods

### 2.1 Protocol and registration

The protocol has been registered on the Open Science Framework (OSF) platform (https://osf.io/dxzhr/), registration number: DOI 10.17605/OSF.IO/DXZHR. This protocol was drafted and reported in accordance with the Preferred Reporting Items for Systematic Reviews and Meta-Analyses Protocols guidelines.^[12]^ If there are any adjustments throughout the study, we will fix and update the details in the final report.

### 2.2 Ethics

We will not need individual data of each patient in the research as this is a systematic review. Therefore institutional review board approval and ethics committee is not needed. Our purpose is to publish the results in a peer-reviewed journal. The final results of the review will provide information about the safety and efficacy of XFZY and its modified forms in the treatment of PD to help clinicians make decisions on clinical practice.

### 2.3 Inclusion criteria

#### 2.3.1 Type of study design

The control group chose broad-spectrum antibiotics according to experience, and the experimental group combined with Shaofu Zhuyu decoction or Jiangfu Zhuyu pills on the basis of the control group. The dosage, dosage and treatment time of the 2 groups were not considered in this study. Studies involving acupuncture, moxibustion, massage, and other TCM prescriptions will be cancelled. In addition, the author will cancel studies involving non-fixed prescriptions of traditional Chinese medicine.

#### 2.3.2 Participants

The patients of PD must meet the diagnostic criteria established by the relevant diagnostic criteria in Obstetrics and Gynecology and the guiding principles for Clinical Research of New drugs of traditional Chinese Medicine.^[11]^ Does not include chronic pelvic inflammation, gynecological malignant tumors, endometriosis, secondary dysmenorrhea, and other gynecological diseases. No gender, race, nationality, and comorbidity are limited.

#### 2.3.3 Interventions

The control group selected analgesic drugs according to experience, while the experimental group was treated with Shaofu Zhuyu pills or modified Shaofu Zhuyu decoction on the basis of the control group. The dose, dose, and treatment time of the 2 groups were not considered in this study. Research on prescriptions of TCM such as acupuncture and massage will be eliminated. In addition, the author is about to cancel studies involving unfixed prescriptions of TCM.

#### 2.3.4 Outcomes

The primary outcomes was evaluated according to the criteria for judging the symptom score of dysmenorrhea, which were formulated with reference to the guiding principles of Clinical Research of New drugs of traditional Chinese Medicine, that is, the basic score was 5 points: dysmenorrhea occurred in perimenstrual period, those who felt obvious pain was marked as 0.5, those who could not bear pain were marked as 1, those who were restless and restless were scored as 1, those with a disgraceful face were scored as 0.5, those with cold limbs were scored as 2, and those with cold sweat were scored as 1. The higher the score, the more serious the symptoms of dysmenorrhea. Efficacy criteria: after treatment, pain disappeared completely, dysmenorrhea symptom score returned to zero, and no recurrence in 3-month menstrual cycle was cured; dysmenorrhea symptom score decreased ≥50% after treatment as significant effect; dysmenorrhea symptom score decreased ≥30% and < 50% was effective after treatment; dysmenorrhea symptom score decreased less than 30% after treatment, or no significant improvement, or increased as invalid. Total clinical effective rate = (number of cured cases + markedly effective cases + effective cases) / total number of people × 100%.

The secondary outcomes included uterine artery pulsation index (arterial pulsatility index,API), resistance index (resistance index,RI), peak systolic/diastolic peak (systolic/diastolic, S/D), serum pain-related mediators and endogenous opioid peptides.

### 2.4 Search methods

#### 2.4.1 Electronic searches

Following databases will be searched: PubMed, MEDLINE, excerpt medica database, Cochrane Library, China National Knowledge Infrastructure, Wanfang data, Chinese Scientific Journals Database (VIP), and China biomedical literature database. We will select the eligible studies published up to May 16, 2021. We adopt the combination of heading terms and free words as search strategy which decided by all the reviewers. Search terms: Shaofu Zhuyu pills, Shaofu Zhuyu decoction, modified Shaofu Zhuyu decoction, dysmenorrhea, primary dysmenorrhea. Taking PubMed as an example, the initial search strategy is shown in Table 1, which will be adjusted according to the specific database.

**Table 1:** Search strategy of the PubMed.

Number	Search terms
#1	Primary dysmenorrhea[Mesh]
#2	Primary dysmenorrhea[Title/Abstract] OR Dysmenorrhea[Title/Abstract] OR The primary dysmenorrhea[Title/Abstract] OR The dysmenorrhea[Title/Abstract]
#3	#1 OR #2
#4	Erchen[Title/Abstract]
#5	Decoction[Title/Abstract]
#6	#4 AND #5
#7	randomized controlled trial[Publication Type]
#8	controlled clinical trial[Publication Type]
#9	randomized[Title/Abstract]
#10	randomly[Title/Abstract]
#11	#10 OR #11 OR #12 OR #13
#12	#3 AND #6 AND #11

#### 2.4.2 Searching other resources

At the same time, we will retrieve other resources to complete the deficiencies of the electronic databases, mainly searching for the clinical trial registries and grey literature about XFZY for PD on the corresponding website.

### 2.5 Data collection and analysis

#### 2.5.1 Selection of studies

**Figure 1:**
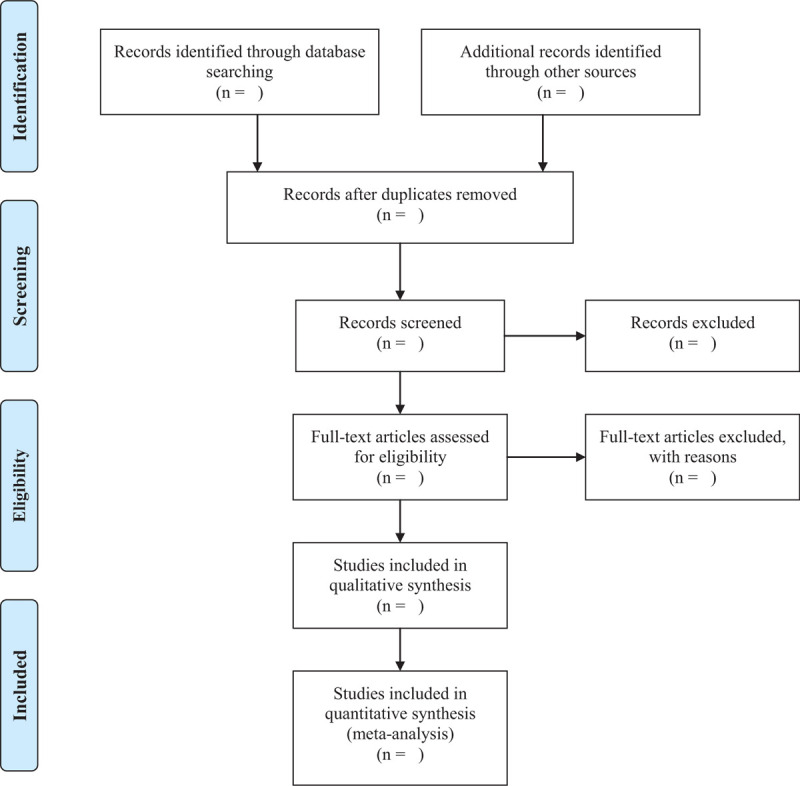
PRISMA flow diagram of the study selection process.

Import all literatures that meet the requirements into Endnote X8 software (Thomson Research Soft, Stanford, Connecticut). First of all, 2 independent reviewers (Yuan Lei, Kang Xueliang) initially screened the literatures that did not meet the pre- established standards of the study by reading the title and abstract. Secondly, download the remaining literatures and read the full text carefully to further decide whether to include or not. Finally, the results wer cross-checked repeatedly by reviewers. If there is a disagreement in the above process, we can reach an agreement by discussing between both reviewers or seek a third party's opinion (Ye Yu). Flow chart of the study selection (Fig. 1) will be used to show the screening process of the study.

#### 2.5.2 Data extraction and management

According to the characteristics of the study, we prepare an excel form for data collection before data extraction. Outcome indicators for eligible studies were independently extracted and filled in the data extraction form by 2 reviewers (Yuan Lei, Kang Xueliang). If there is any argument, it can get an agreement by discussing through 2 reviewers or seek a third party's suggestion (Ye Yu). The main data extracted are as follows: title, author, year, fund source, sample size, age, sex, duration of disease, interventions, outcome measures, adverse reactions, etc. If you find something unclear in the study, you can contact the author of the communication directly for more detailed information. The above information was finally cross-checked by 2 reviewers.

#### 2.5.3 Assessment of risk of bias in included studies

The quality assessment of randomized controlled trials adopts the risk of bias (ROB) assessment tool provided by the Cochrane Handbook. The following 7 items, such as random sequence generation, allocation concealment, blinding of participants and personnel, blinding of outcome assessment, incomplete outcome data, selective outcome reporting, and other bias, are evaluated by 3 grades of “low bias,” “high bias,” and “unclear bias.” The discrepancies will get a consistent conclusion by discussing between both reviewers or seeking the third-party consultation.

#### 2.5.4 Measures of treatment effect

Different evaluation methods are selected according to the different efficacy indicators. For the dichotomous data, we will choose the effect scale indicator relative risk with 95% confidence interval to represent. While the continuous data is expressed as mean difference or standardized mean difference with 95% confidence interval depending on whether the measurement scale is consistent or not.

#### 2.5.5 Dealing with missing data

The reviewers will contact the first author or correspondent author via email or telephone to obtain missing data if the relevant data is incomplete. If the missing data is still not obtained in the above way, we can synthesize the available data in the initial analysis. Furthermore, sensitivity analysis will be used to assess the potential impact of missing data on the overall results of the study.

#### 2.5.6 Assessment of heterogeneity

Heterogeneity will be assessed by Chi-Squared test and *I*^2^ test. If *I*^2^ < 50%, *P* > .1, we consider that no statistical heterogeneity between each studies and choose fixed effect model to synthesize the data. If *I*^2^ ≥ 50%, *P* < .1, indicating that there is a statistical heterogeneity, the data are integrated by the random effect model. In addition, due to differences in heterogeneity, we will conduct subgroup or sensitivity analysis to look for the potential causes.

#### 2.5.7 Data analysis

Review Manager software version 5.3 (The Nordic Cochrane Center, The Cochrane Collaboration, 2014, Copenhagen, Denmark) provided by the Cochrane Collaboration will be performed for data synthesis and analysis. The dichotomous data is represented by RR, continuous data is expressed by mean difference or standardized mean difference. If there is no heterogeneity (*I*^2^ < 50%, *P* > .1),the data are synthesized using a fixed effect model. Otherwise (*I*^2^ ≥ 50%, *P* < .1), a random effect model is used to analyze. Then subgroup analysis will be conducted basing on the different causes of heterogeneity. If a meta-analysis cannot be performed, it will be replaced by a general descriptive analysis.

#### 2.5.8 Subgroup analysis

If the results of the study are heterogeneous, we will conduct a subgroup analysis for different reasons. Heterogeneity is manifested in the following several aspects, such as race, age, sex, different intervention forms, pharmaceutical dosage form, dosage, treatment course.

#### 2.5.9 Sensitivity analysis

Sensitivity analysis is mainly used to evaluate the robustness of the primary outcome measures. The method is that removing the low-level quality study one by one and then merge the data to assess the impact of sample size, study quality, statistical method, and missing data on results of meta-analysis.

#### 2.5.10 Reporting bias

If there are >10 studies in the meta- analysis, the symmetry of the funnel plot will be assessed to examine publication bias, with results being interpreted cautiously.

#### 2.5.11 Grading the quality of evidence

In this systematic review, the quality of evidence for the entire study is assessed using the “Grades of Recommendations Assessment, Development and Evaluation (GRADE)” standard established by the World Health Organization and international organizations.^[12]^ To achieve transparency and simplification, the GRADE system divides the quality of evidence into 4 levels: high, medium, low, and very low. The GRADE profiler 3.2 will be employed for analysis.

## 3 Discussions

Primary dysmenorrhea is a common and frequently-occurring disease in women, which is easy to cause some women's menstrual anxiety and seriously perplexes women's study and life.^[1,13,14]^ At present, Western medicine aims at relieving pain, although the effect is obvious, but there is no effective and thorough cure, dispel the cause of the disease, can not achieve the expected effect. According to traditional Chinese medicine, the treatment of primary dysmenorrhea should promote blood circulation and remove blood stasis, dredge menstruation and relieve pain, so Shao Fu Zhuyu Pill is selected to treat it.^[15,16]^ A number of clinical studies at home and abroad have shown that the application of Shaofu Zhuyu pills in the treatment of primary dysmenorrhea can significantly improve the pain symptoms and improve the therapeutic effect. Therefore, we conducted a systematic review to further evaluate the efficacy and safety of Xuefu Zhuyu decoction in the treatment of primary dysmenorrhea. Our aim is to provide more clinical evidence to help clinicians make decisions in the clinical practice of PD treatment.

## Author contributions

**Conceptualization:** Lei Yuan, XueLiang Kang.

**Data curation:** XueLiang Kang.

**Formal analysis:** XueLiang Kang.

**Funding acquisition:** Yu Ye.

**Investigation:** Dan Lin.

**Methodology:** Dan Lin.

**Project administration:** Yu Ye.

**Supervision:** XueLiang Kang.

**Writing – original draft:** Lei Yuan.

**Writing – review & editing:** Lei Yuan.
